# Supplementary material for: Fibroblast growth factor 21 increases insulin sensitivity through specific expansion of subcutaneous fat
Source: Nat Commun. 2018 Jan 18;9:272. doi: 10.1038/s41467-017-02677-9 (PMC5773530; doi:10.1038/s41467-017-02677-9)
Supplement: Supplementary file 2 — Supplementary Information [file 41467_2017_2677_MOESM2_ESM.pdf]

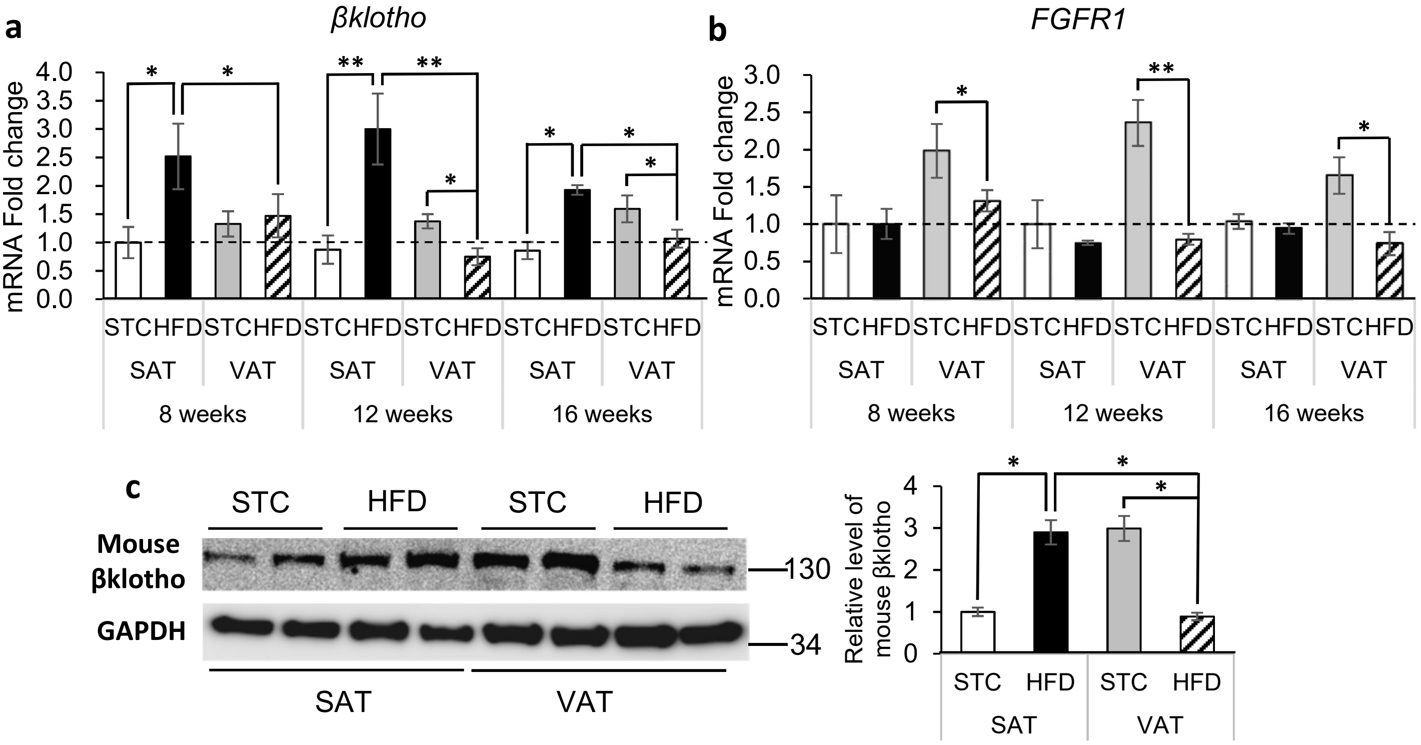

Supplementary Figure 1. FGF21 coreceptor βklotho exhibits fat depot-difference in HFD-induced mice. (**a**) Change of *βklotho* expression during STC or HFD induction in SAT and VAT of WT mice. All data were normalized to *βklotho* expression in SAT of mice fed on STC. n = 10-12. (**b**) Change of *FGFR1* expression during STC or HFD induction in SAT and VAT of WT mice. All data were normalized to *FGFR1* expression in SAT of mice fed with STC. n = 10-12. (**c**) Western blot analysis and densitometric quantification of mouse βklotho protein levels in SAT and VAT of WT mice fed with STC or HFD for 8 weeks. Data are presented as mean ± s.e.m. Significance was determined by one-way ANOVA with Bonferroni multiple-comparison analysis. * $P < 0.05$, ** $P < 0.01$, *** $P < 0.001$.

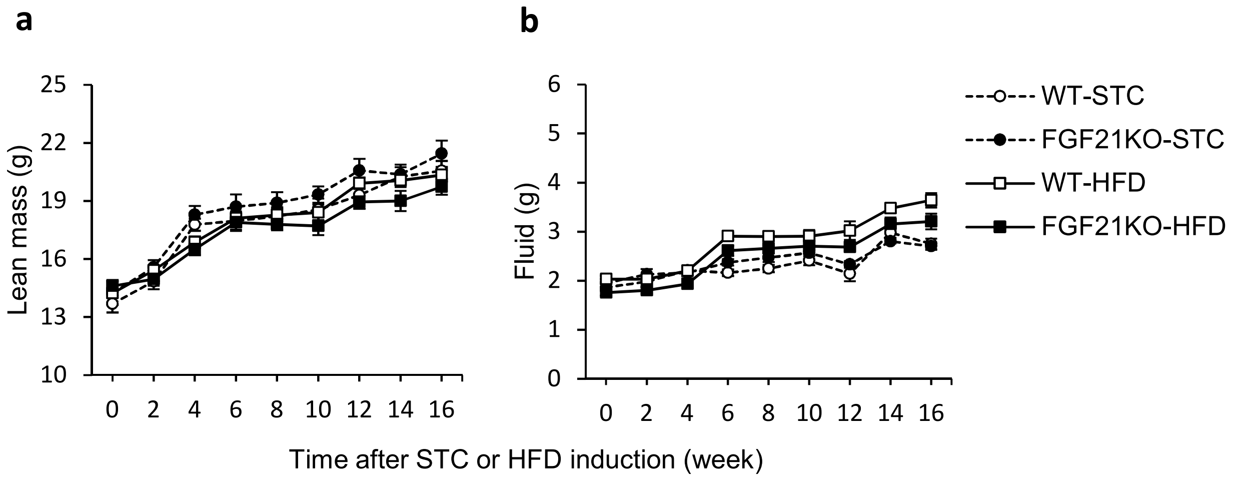

Supplementary Figure 2. Body composition in FGF21KO and WT mice during STC or HFD induction.
Eight-week-old, male FGF21KO and WT mice were fed with STC or HFD for 16 weeks. No significances of (**a**) Lean mass and (**b**) fluid were found between FGF21KO and WT fed with either diet. n = 10-12. Data are presented as mean ± s.e.m. Significance was determined by two-way ANOVA with Bonferroni multiple-comparison analysis.

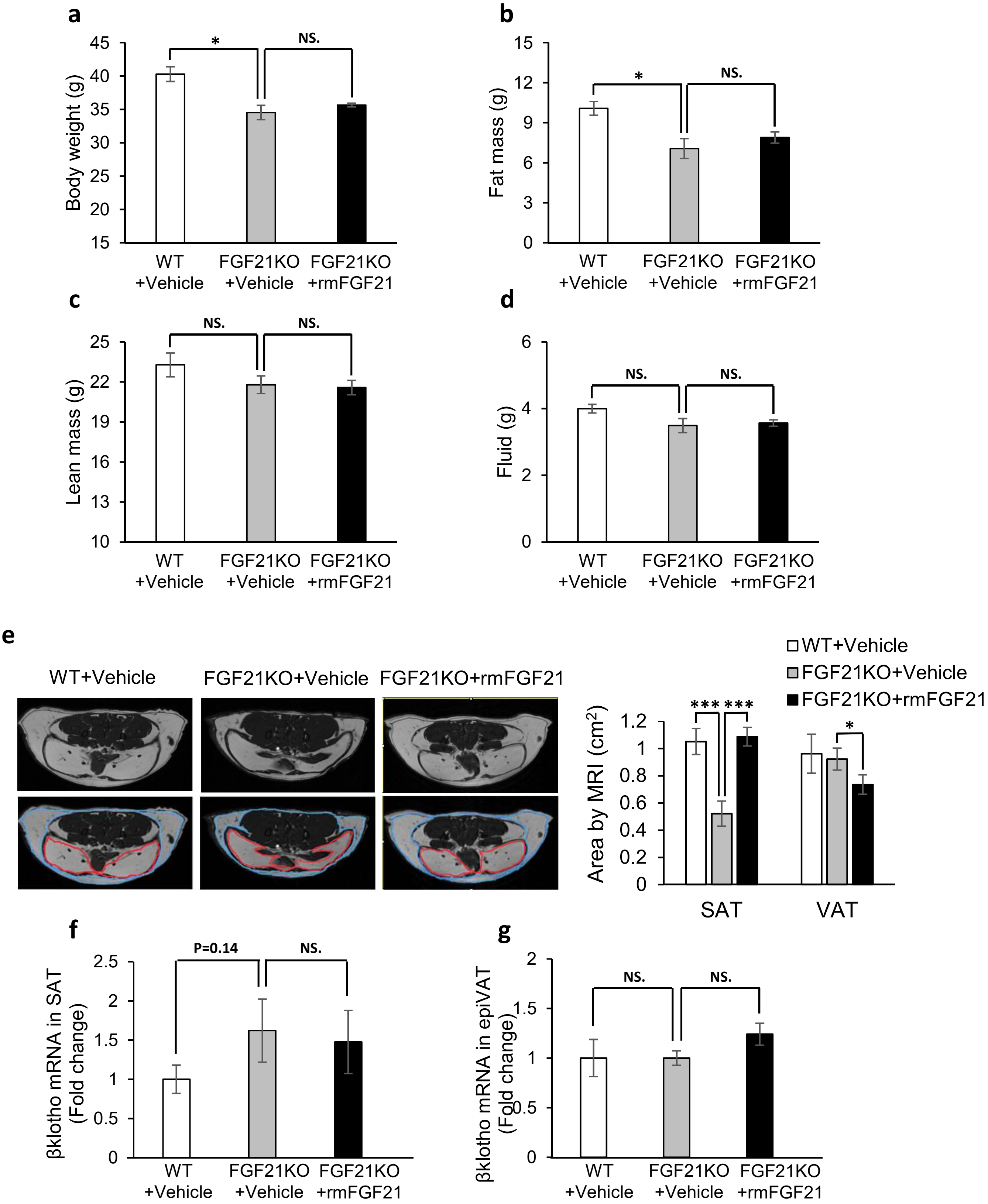

Supplementary Figure 3. Replenishment with physiological dose of rmFGF21 in FGF21KO mice alters fat distribution measured by MRI and does not alter their body weight and net weight of fat mass.

(**a**) Body weight, (**b**) fat mass, (**c**) lean mass, (**d**) fluid, in WT+Vehicle, FGF21KO+Vehicle and FGF21KO+rmFGF21 groups after 4 weeks' intervention. (**e**) Effect of chronic rmFGF21 treatment to HFD-induced physiological level on global SAT and VAT as measured by MRI and quantified by Omics software. Raw (upper panel) and marked MRI (lower panel), blue lines delineate SAT and red lines delineate VAT. (**f**) βklotho expression level in SAT and (**g**) βklotho expression level in epiVAT in WT+Vehicle, FGF21KO+Vehicle and FGF21KO+rmFGF21 groups after 4 weeks' intervention. Data were normalized to βklotho level of WT+Vehicle mice. n = 6. These results were reproduced in four independent experiments. Data are presented as mean ± s.e.m. Significance was determined by one-way ANOVA with Bonferroni multiple-comparison analysis. *, $P < 0.05$, ** $P < 0.01$, *** $P < 0.001$, NS., non-significance.

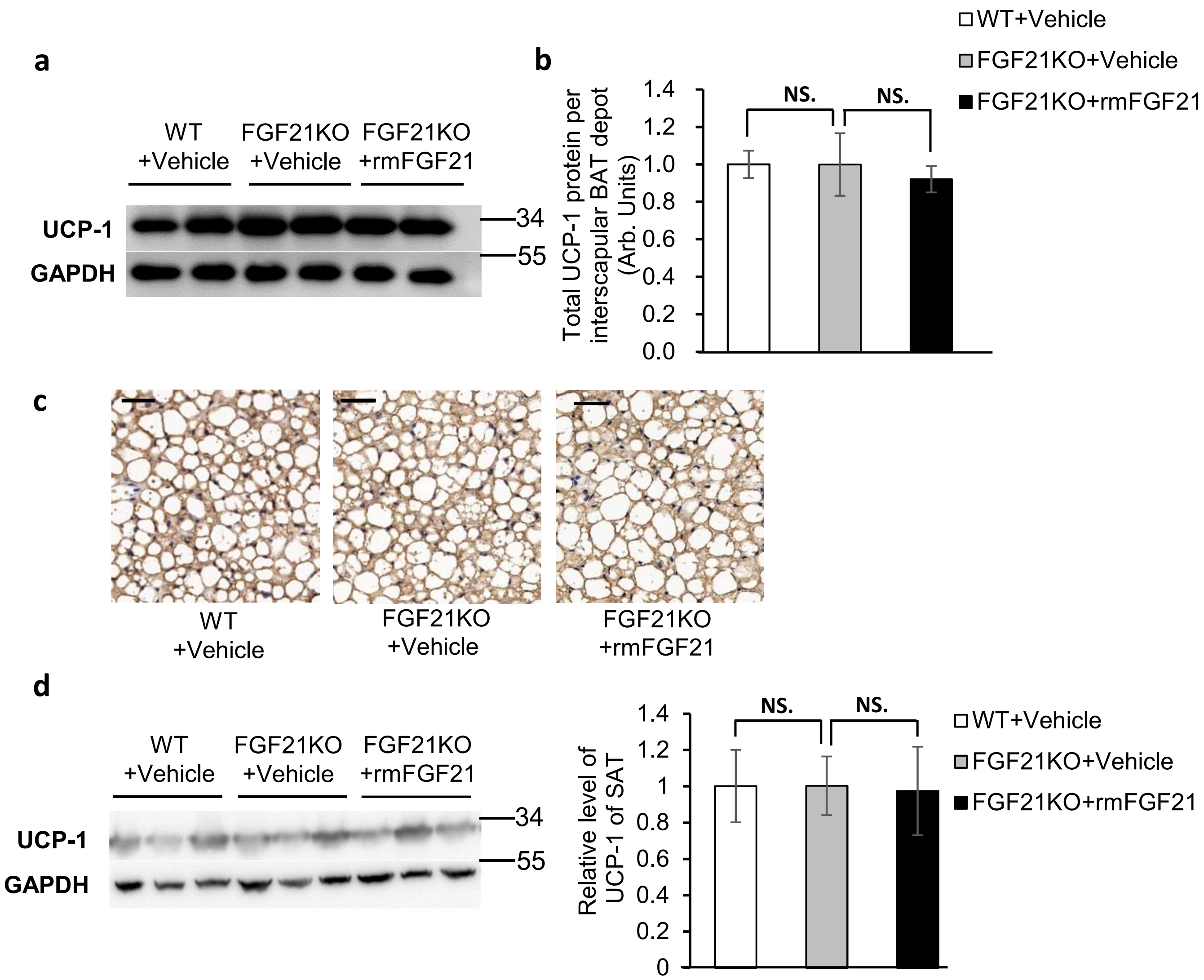

Supplementary Figure 4. Treatment with physiological level of rmFGF21 does not cause change of BAT activity or browning of SAT.

(**a**) Western blot analysis of mouse relative UCP-1 protein levels in BAT of WT+Vehicle, FGF21KO+Vehicle and FGF21KO+rmFGF21 groups after HFD induction. n = 5. (**b**) Total UCP-1 protein amount in the interscapular BAT depot calculated from densitometric quantification of relative UCP-1 protein levels and total homogenate protein in the interscapular BAT depot. (**c**) Representative figure of immunohistochemistry staining of UCP-1 of BAT in three groups. Scale bar = 50μm. n = 5. (**d**) Western blot analysis and densitometric quantification of relative mouse UCP-1 protein levels in SAT of WT+Vehicle, FGF21KO+Vehicle and FGF21KO+rmFGF21 groups after HFD induction. n = 5. Data are presented as mean ± s.e.m. Significance was determined by Student's *t* test.

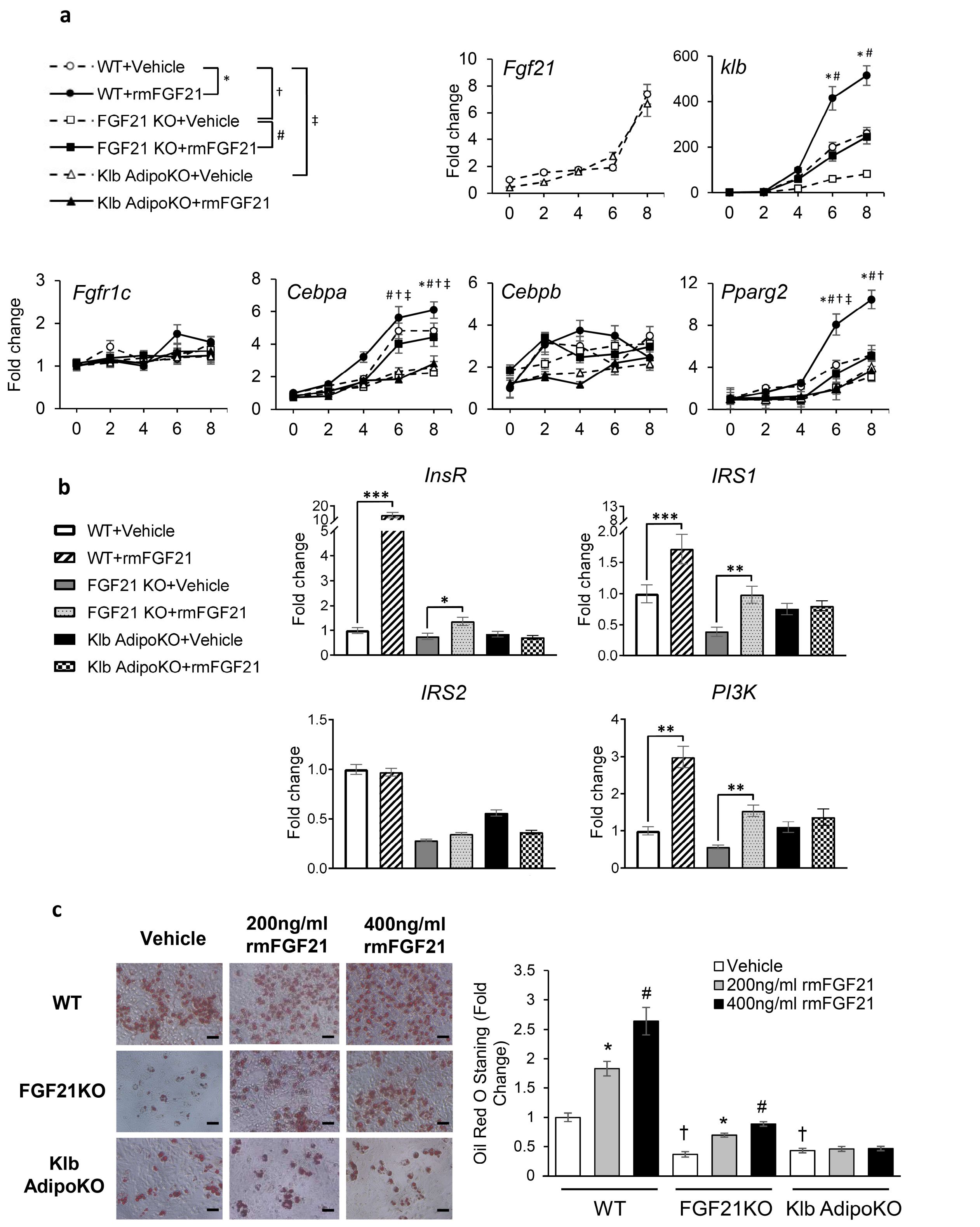

Supplementary Figure 5. FGF21KO and Klb AdipoKO adipocytes have altered adipogenic capacity and insulin sensitivity.

SVF preadipocytes isolated from SAT of WT, FGF21KO and Klb AdipoKO mice were differentiated in vitro over an 8-day period. These preadipocytes were differentiated in either the presence or absence of rmFGF21 (200 or 400 ng/ml). n = 6 wells. These results were reproduced in three independent experiments. (**a**) Gene expression in 8-day period of 400 ng/ml rmFGF21 treatment measured by RT-qPCR. *, p<0.05, WT+Vehicle *vs.* WT+rmFGF21. #, p<0.05, FGF21KO+Vehicle *vs.* FGF21KO+rmFGF21. †, p<0.05, FGF21KO+Vehicle *vs.* WT+Vehicle. ‡, p<0.05, Klb AdipoKO+Vehicle *vs.* WT+Vehicle. (**b**) Gene expression of differentiated adipocytes treated with 400ng/ml rmFGF21. * $P < 0.05$, ** $P < 0.01$, ***, $P < 0.001$. (**c**) Lipid accumulation was measured by oil red O staining. Representative oil red O-stained cells at 8th day of differentiation are shown. Scale bar = 50μM. *vs. mice treated with vehicle from the same group, #vs. mice treated with 200ng/ml rmFGF21 from the same group, †vs. WT mice treated with vehicle. Data are presented as mean ± s.e.m. Significance was determined by one-way ANOVA (**b,c**) and two-way ANOVA (**a**) with Bonferroni multiple-comparison analysis.

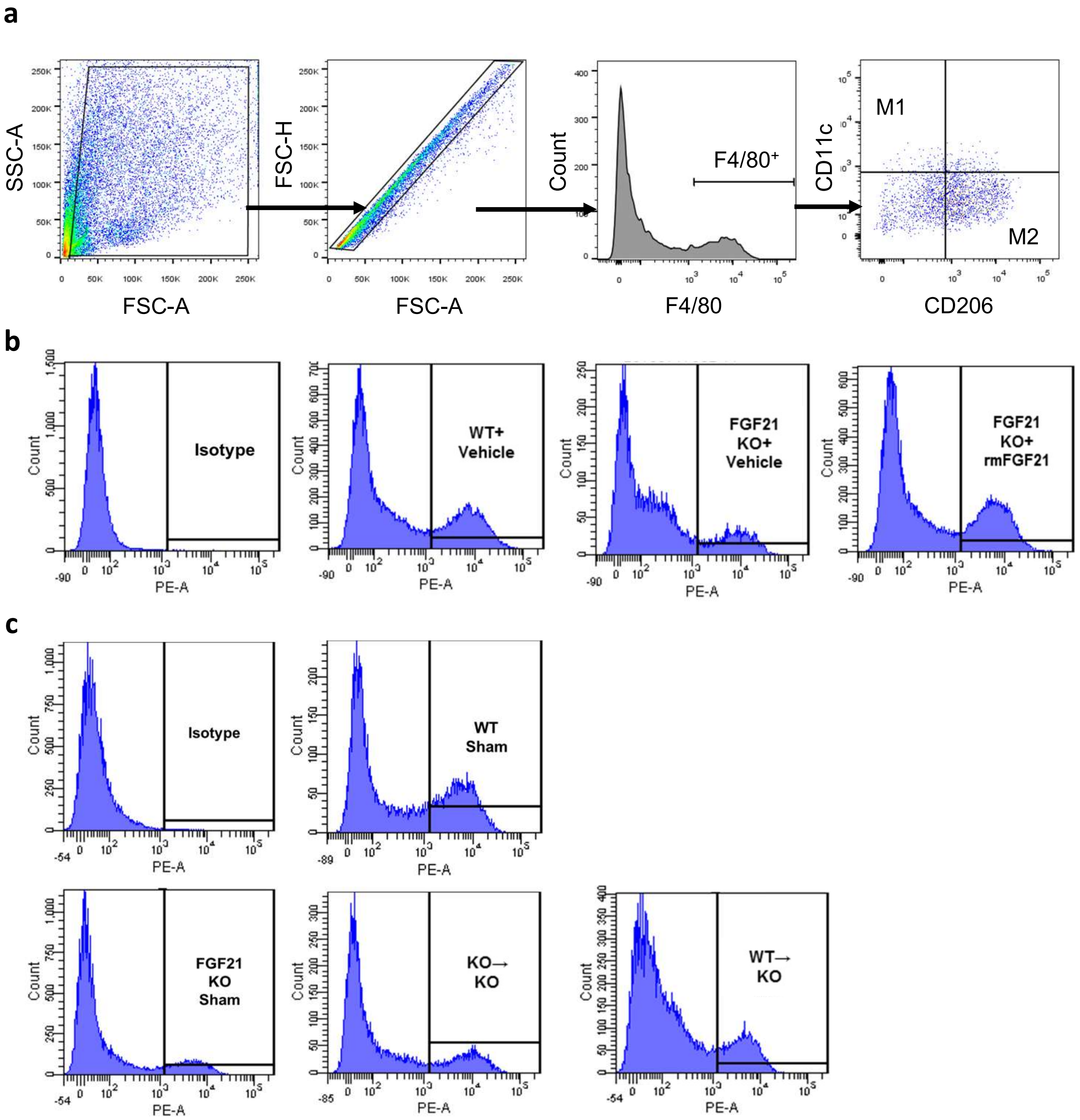

Supplementary Figure 6. Representative plot chats of flow cytometry.

(**a**) Gating strategy of total, M1 and M2 macrophage (with example plots). (**b**) Flow cytometry analyzing F4/80^{+} cells in WT+Vehicle, FGF21KO+Vehicle and FGF21KO+rmFGF21 groups. (**c**) Flow cytometry analyzing F4/80^{+} cells in WT Sham, KO Sham, KO→KO and WT→KO groups.

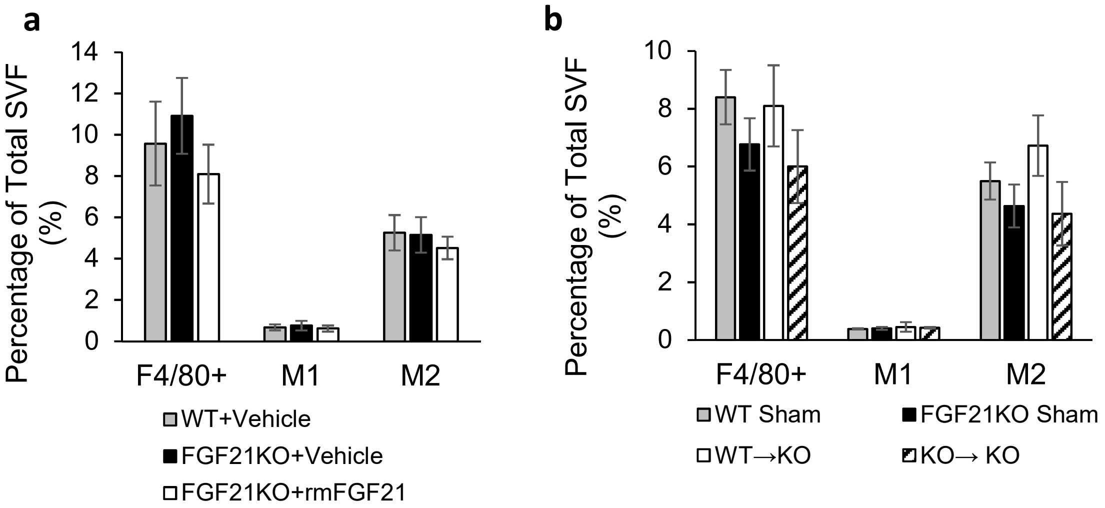

Supplementary Figure 7. FGF21 has no obvious effect on M2 macrophage polarization in visceral adipose tissue. Flow cytometry analysis for total, M1 and M2 macrophages in SVF of VAT in (**a**) WT+Vehicle, FGF21KO+Vehicle and FGF21KO+rmFGF21 groups, (**b**) WT Sham, KO Sham, KO→KO and WT→KO groups. n = 6. These results were reproduced in two independent experiments. Data are presented as mean ± s.e.m.

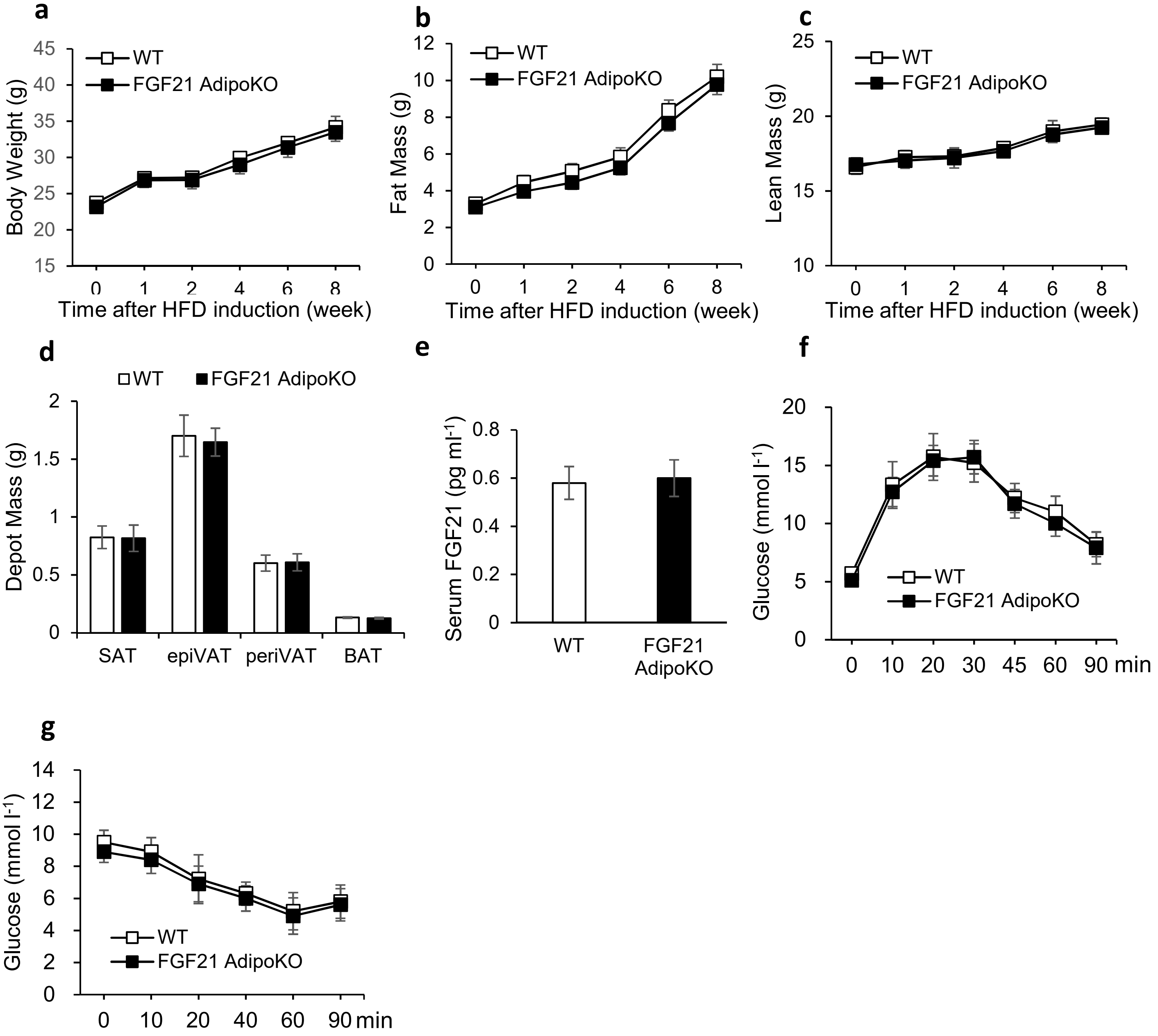

Supplementary Figure 8. Adipose tissue specific FGF21-knockout mice (FGF21 AdipoKO) did not have decreased fat mass and glucose intolerance after HFD induction.

Eight-week-old, male WT and FGF21 AdipoKO mice were fed with HFD for 8 weeks. n=8/group. (**a-c**) (**a**) Body weight, (**b**) fat mass and (**c**) lean mass were measured at various time periods. (**d**) Adipose tissue mass was measured for SAT, epiVAT, periVAT and BAT depots in WT and FGF21 AdipoKO mice fed with HFD. (**e**) Serum FGF21 levels at fed state in WT and FGF21 AdipoKO mice during HFD induction were measured by ELISA. (**f,g**) (**f**) Glucose tolerance test (GTT) performed in WT and FGF21 AdipoKO mice fed with HFD for 8 weeks and (**g**) Insulin tolerance test (ITT) performed in WT and FGF21 AdipoKO mice fed with HFD for 8 weeks. Data are presented as mean ± SEM. Significance was determined by student's *t* test. * $p<0.05$.

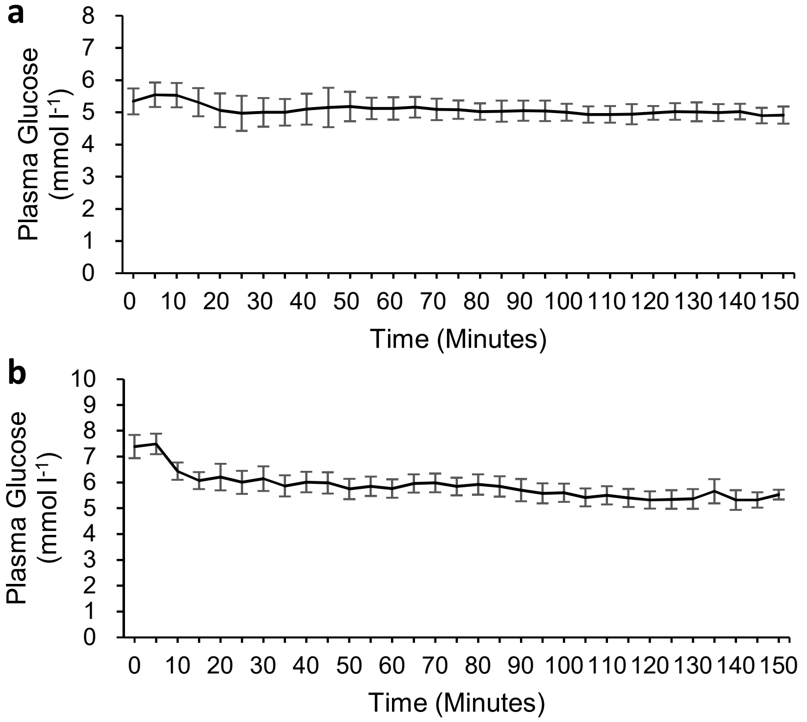

Supplementary Figure 9. Summary of plasma glucose levels during hyperinsulinemic-euglycemic clamp.
(**a**) Summary of plasma glucose levels during hyperinsulinemic-euglycemic clamp on human, data are presented as mean ± s.d. (**b**) Summary of plasma glucose levels during hyperinsulinemic-euglycemic clamp on mice, data are presented as mean ± s.e.m.

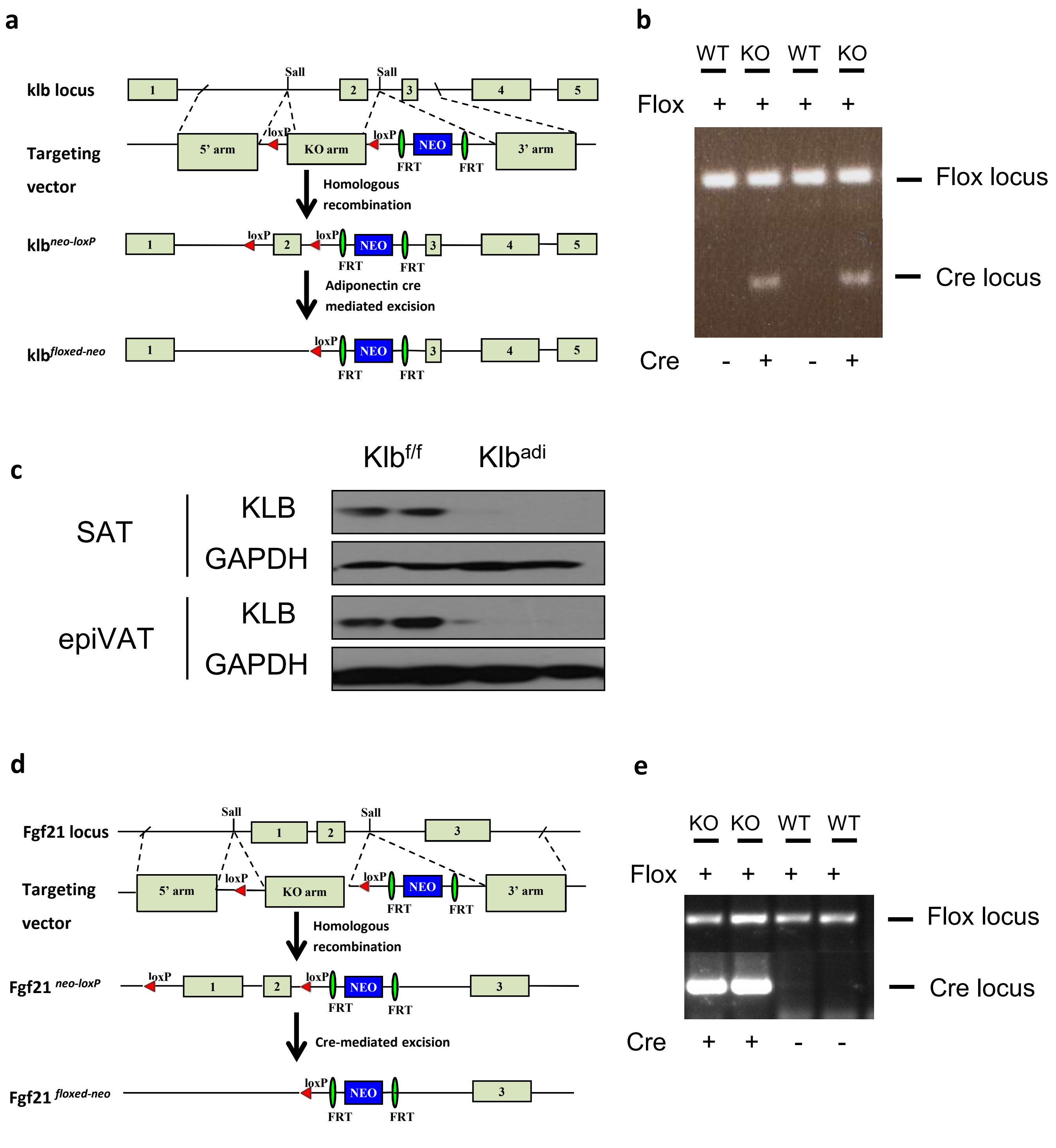

Supplementary Figure 10. Adipose-selective ablation of βKlotho or FGF21.
(**a**) Generation of mice with adipose-selective ablation of βKlotho (Klb AdipoKO). (**b**) Genotyping of Klb AdipoKO mice by genomic PCR. (**c**) Western blot of βKlotho in SAT and epiVAT of WT and Klb AdipoKO mice. (**d**) Generation of mice with adipose-selective ablation of FGF21 (FGF21 AdipoKO). (**e**) Genotyping of FGF21 AdipoKO mice by genomic PCR.

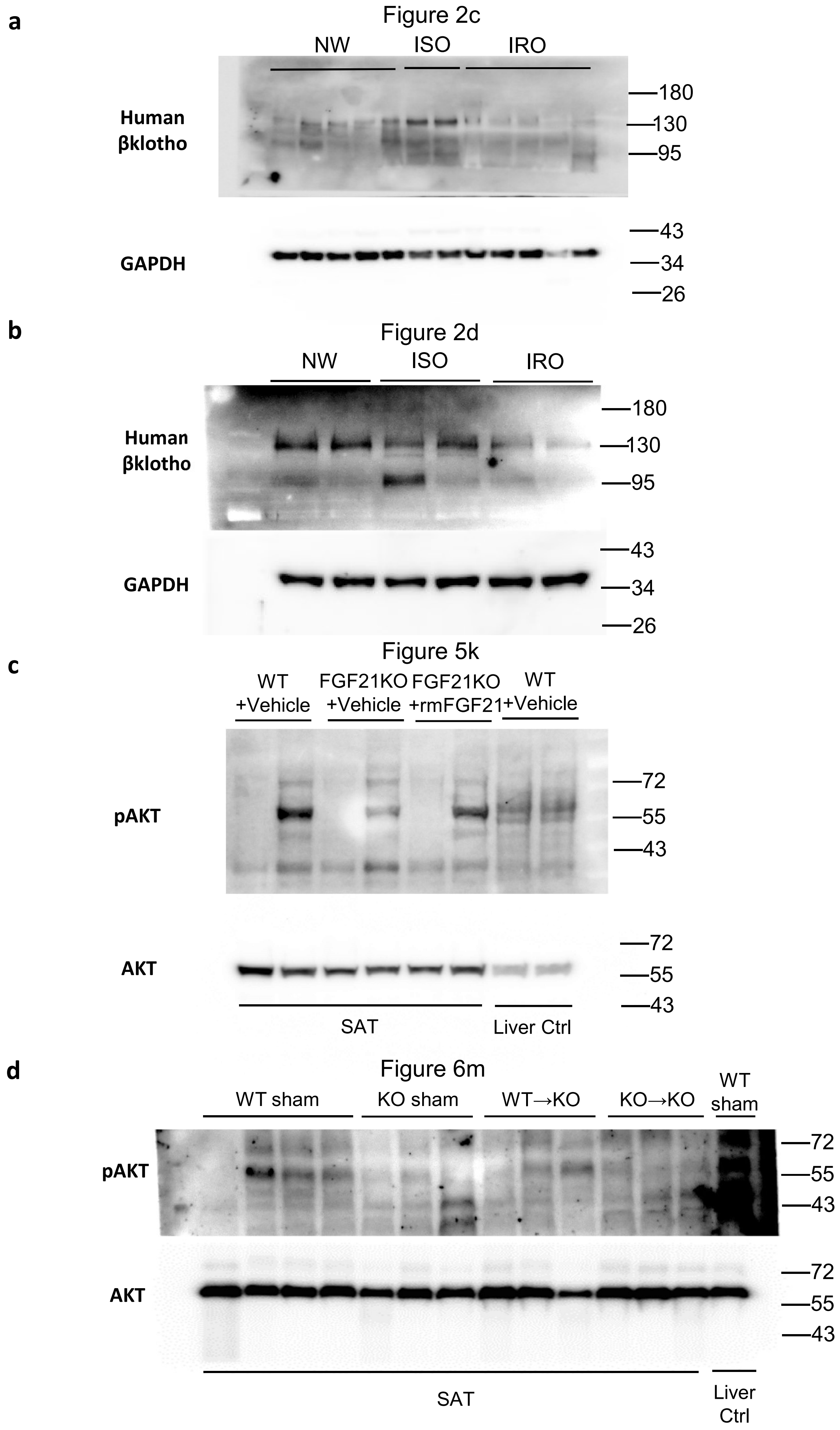

Supplementary Figure 11. Uncropped scans of the immunoblots for Figure 2, 5, 6.

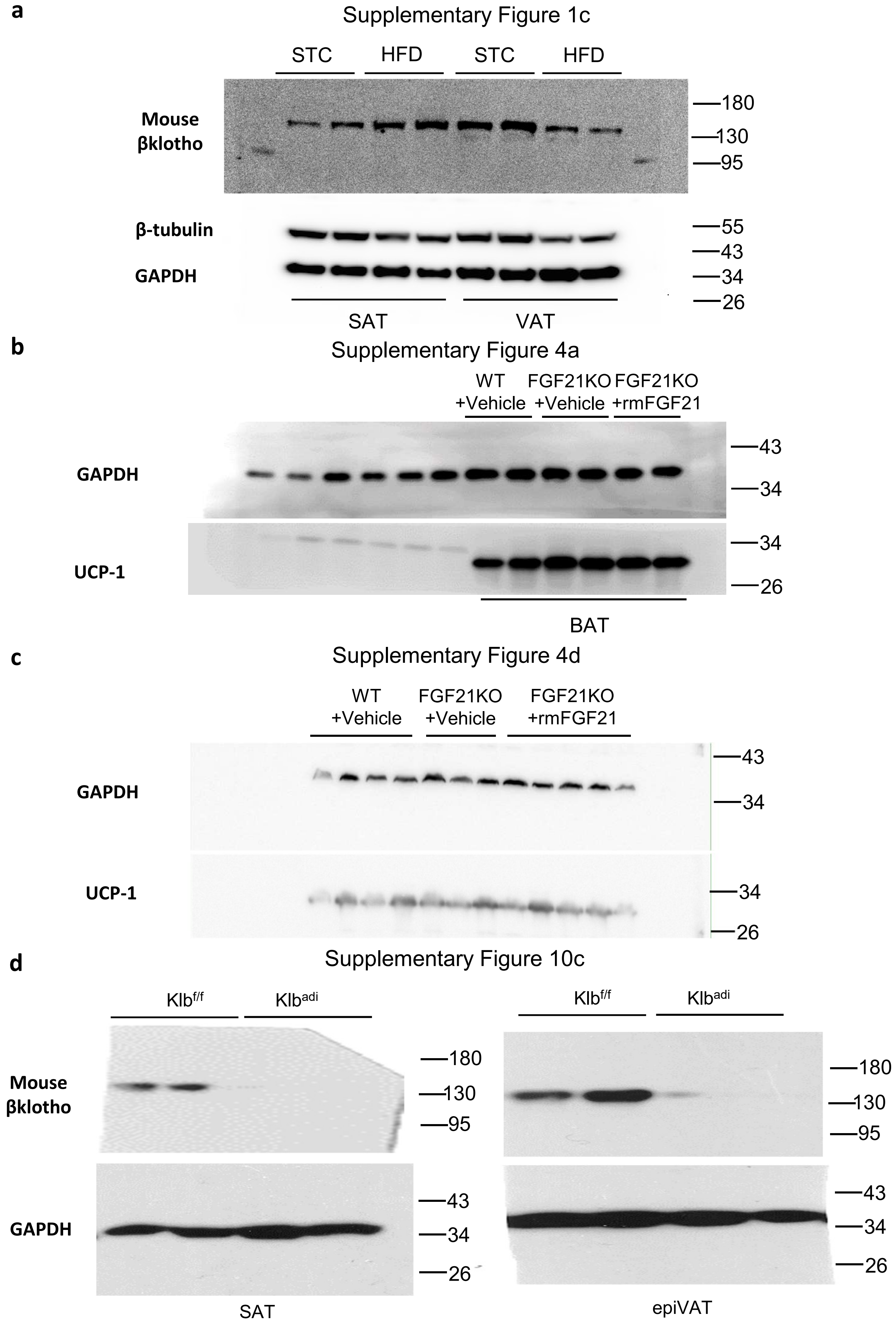

Supplementary Figure 12. Uncropped scans of the immunoblots for Supplementary Figure 1, 4, 10.

Supplementary Table 1. Anthropometric and biochemical parameters of the study subjects.

Variables	NW (n = 30)	ISO (n = 30)	IRO (n = 30)
Male/Female	18/12	18/12	19/11
Age (years)	32.10±2.52	32.03±4.98	34.00±4.90
BMI (kg m^{-2})	20.90±1.70	27.53±1.95***	28.18±2.14***
Total fat mass (kg)	12.01±3.60	23.69±6.20***	24.58±6.88***
Total fat percentage (%) §	22.60 (16.30, 26.95)	26.50 (24.68, 38.20)***	28.70 (26.25, 37.50)***
FPG (mmol l^{-1})	4.86±0.38	4.92±0.35	5.25±0.41***##
2hPG (mmol l^{-1})	5.84±0.83	6.33±1.04	6.70±1.41**
FINS (mU l^{-1}) §	7.69 (5.56, 11.28)	8.34 (6.70, 10.83)	14.59 (11.44, 18.50)***###
2hINS (mU l^{-1}) §	33.06 (21.76, 43.03)	37.79 (27.17, 55.17)	51.77 (40.87, 86.35)***##
HOMA-IR §	1.62 (1.16, 2.57)	1.78 (1.37, 2.32)	3.55 (2.75, 3.97)***###
Waist circumference (cm)	71.40±5.93	89.51±5.09***	95.63±8.50***##
SFA (cm^2) §	131.00 (93.99, 142.52)	256.09 (217.11, 294.15)***	224.75 (163.82, 278.35)***#
VFA (cm^2) §	30.30 (23.09, 36.74)	51.69 (37.47, 92.68)***	114.66 (91.78, 144.23)***##
SFA/VFA §	3.09 (3.03, 4.83)	5.20 (4.04, 7.41)*	2.15 (1.34, 3.01)*##
SBP (mmHg)	110.38±12.88	109.67±12.17	112.12±9.35
DBP (mmHg)	69.92±9.42	73.93±6.71	75.40±6.16
TC (mmol l^{-1})	4.36±0.74	4.32±0.55	4.68±0.80#
TG (mmol l^{-1}) §	0.78 (0.63, 1.01)	0.99 (0.73, 1.22)*	1.01 (0.79, 1.44)*
HDL-C (mmol l^{-1})	1.27 (1.17, 1.40)	1.09 (0.93, 1.27)**	1.14 (0.87, 1.31)*
LDL-C (mmol l^{-1})	2.63±0.56	2.80±0.41	3.09±0.67**#
GIR (mg kg^{-1} min^{-1}) §	7.91 (6.23, 9.63)	6.62 (5.24, 8.05)	4.05 (3.22, 6.12)*#
Adiponectin (ug ml^{-1}) §	10.65 (8.32, 16.03)	11.01 (8.58, 13.57)	9.36 (7.67, 12.14)*
FGF21 (pg ml^{-1}) §	79.05 (52.62, 136.28)	194.66 (119.66, 256.86)***	134.27 (88.95, 207.78)**#

Data are means ± SD or median (interquartile range). § Log transformed before analysis.
vs. NW, * P<0.05, ** P<0.01, *** P<0.001; ISO vs. IRO, # P<0.05, ## P<0.01, ### P<0.001.
FPG, fasting plasma glucose; 2hPG, 2-hour plasma glucose during OGTT; FINS, fasting insulin; 2hINS, 2-hour insulin during OGTT; SBP, systolic blood pressure; DBP, diastolic blood pressure; TC, total cholesterol; TG, triglyceride.

Supplementary Table 2. Sequence of the RT-qPCR primers.

Name	Forward	Reverse
Human		
βklotho	CCATCCGCCGAGGATTATTT	GGTAGTGGGTGACTTTCATTCT
FGFR1	CCCGTAGCTCCATATTGGACA	TTTGCCATTTTTCAACCAGCG
18s	CAATTACAGGGCCTCGAAAG	AAACGGCTACCACATCCAAG
Mouse		
Adiponectin	GGAGAGAAAGGAGATGCAGGT	CTTTCCTGCCAGGGGTTC
Arg1	TGGCTTGCGAGACGTAGAC	GCTCAGGTGAATCGGCCTTTT
βklotho	TGGTTCGCCAACCCCATCCA	TGGGCCCGAAGGAAAAGGCA
Cd11c	CTGGATAGCCTTTCTTCTGCTG	GCACACTGTGTCCGAACTC
Cebpa	GAGTCGGCCGACTTCTACG	GTCTCGTGCTCGCAGATGC
FGFR1c	GCCAGACAACTTGCCGTATG	ATTTCCTTGTCGGTGGTATTAACTC
F4/80	CTTTGGCTATGGGCTTCCAGTC	GCAAGGAGGACAGAGTTTATCGTG
Gapdh	GACGGCCGCATCTTCTTGT	CACACCGACCTTCACCATTTT
Glut1	GCCCCCAGAAGGTTATTGA	CGTGGTGA GTGTGGTGGAT
Glut4	CATTCCCTGGTTCATTGTGG	GAAGACGTAAGGACCCATAGC
IL1β	CTGGTGTGTGACGTTCCCATTA	CCGACAGCACGAGGCTTT
IL6	CTGCAAGAGACTTCCATCCAG	AGTGGTATAGACAGGTCTGTTGG
IL10	GCTCTTACTGACTGGCATGAG	CGCAGCTCTAGGAGCATGTG
InsR	GACATCCGGAACAACCTGAC	TCAGCTGTGCAGCCATGTGAC
IRS1	GACTACATGACCATGGACATAG	CGAGTAGGTGCTGAGAAGGTC
IRS2	GTCCAGGCACTGGAGCTTT	GCTGGTAGCGCTTCACTCTT
MCP1	CCACTCACCTGCTGCTACTCA	TGGTGATCCTCTTGTAGCTCTCC
Mgl1	TGAGAAAGGCTTTAAGAACTGGG	GACCACCTGTAGTGATGTGGG
Mrc2	TACAGCTCCACGCTATGGATT	CACTCTCCCAGTTGAGGTACT
PI3Kp85α	AGGGAAGAGGTGAATGAGAG	TTGGACACAGGGTAGAGAAG
Srebf1a	TAGTCCGAAGCCGGGTGGGCGCC GGCGCCAT	GATGTCGTTCAAAACCGCTGTGTGT CCAGTTC
Srebf1c	ATCGGCGCGGAAGCTGTCGGGGT AGCGTC	ACTGTCTTGGTTGTTGATGAGCTGG AGCAT
TNFα	ACGGCATGGATCTCAAAGAC	AGATAGCAAATCGGCTGACG
